# A Comparison of the Chemical Composition, In Vitro Bioaccessibility and Antioxidant Activity of Phenolic Compounds from Rice Bran and Its Dietary Fibres

**DOI:** 10.3390/molecules23010202

**Published:** 2018-01-18

**Authors:** Guanghe Zhao, Ruifen Zhang, Lihong Dong, Fei Huang, Lei Liu, Yuanyuan Deng, Yongxuan Ma, Yan Zhang, Zhencheng Wei, Juan Xiao, Mingwei Zhang

**Affiliations:** 1 Sericultural & Agri-Food Research Institute, Guangdong Academy of Agricultural Sciences/Key Laboratory of Functional Foods, Ministry of Agriculture/Guangdong Key Laboratory of Agricultural Products Processing, Guangzhou 510610, China; greatriver2007@163.com (G.Z.); dolify@163.com (L.D.); hf1311@163.com (F.H.); liulei309@tom.com (L.L.); yuanyuan_deng@yeah.net (Y.D.); mayongxuan@yahoo.cn (Y.M.); zhang__yan_@126.com (Y.Z.); zhencheng_wei@163.com (Z.W.); xiaojuan209218@163.com (J.X.); 2 College of Food Science & Technology, Huazhong Agricultural University, Wuhan 430070, China

**Keywords:** rice bran, dietary fibre, phenolic compounds, antioxidant activity, bioaccessibility

## Abstract

The composition, in vitro bioaccessibility and antioxidant activities of the phenolic compounds in defatted rice bran (DRB) and its soluble and insoluble dietary fibres were systematically evaluated in this study. The total phenolic content of insoluble dietary fibre from DRB (IDFDRB) was much higher than that of the soluble dietary fibre from DRB (SDFDRB) but was 10% lower than that of DRB. Bound phenolics accounted for more than 90% of the total phenolics in IDFDRB, whereas they accounted for 34.2% and 40.5% of the total phenolics in DRB and SDFDRB, respectively. Additionally, the phenolic profiles and antioxidant activities were significantly different in DRB, SDFDRB and IDFDRB. The phenolic compounds in IDFDRB were much less bioaccessibility than those in DRB and SDFDRB due to the higher proportion of bound phenolics in IDFDRB. Considering that bound phenolics could be released from food matrices by bacterial enzymes in the large intestine and go on to exert significant beneficial health effects in vivo, further studies on IDFDRB are needed to investigate the release of the phenolics from IDFDRB via gut microbiota and the related health benefits.

## 1. Introduction

Many studies in humans and experimental models have demonstrated the numerous health benefits associated with an increased intake of dietary fibre. These benefits include regulating body weight and insulin sensitivity, reducing the risk of coronary heart disease, alleviating metabolic syndrome, improving large bowel function, and preventing some cancers [1,2,3]. These health benefits may be closely related to the bioactive components bound to the dietary fibre, especially the active small molecule components such as phenolic compounds [4,5,6].

Recent reports have shown that bound phenolics, which are those phenolics that are mainly bound to the fibre, may give dietary fibre a range of physiological functions. Long-term intake of grape dietary fibre, which is rich in proanthocyanidins, improved lipid profiles and helped control blood pressure in adults [7], and acute intake of this kind of dietary fibre also increased the plasma antioxidant capacity of volunteers [5]. The consumption of carob fibre, which is rich in bound phenolics, lowered total and low density lipoprotein cholesterol in hypercholesterolemic subjects [8]. Furthermore, a diet containing cocoa fibre, which is also rich in bound phenolics, improved lipid profiles and reduced lipid peroxidation in hypercholesterolemic rats [9].

Fresh fruits and vegetables are usually thought to be rich in phenolic compounds, however, the total phenolic contents of whole grains are comparable to those of fruits and vegetables [10]. According to previous reports, most of the phenolics in whole grains distribute in the bran fractions [10,11,12], which are mostly constituted by dietary fibres (for example, accounting for more than 30% in rice bran). Furthermore, a higher proportion of the phenolic compounds is covalently bound to fibre matrix, compared with other matrixs in whole grains [10,11]. Therefore, we can deduce that the dietary fibre of rice bran is an important carrier of phenolics (especially bound phenolics) in rice.

The chemical composition and antioxidant activities of phenolics in brown rice and rice bran [13,14,15,16,17,18,19] and the influences of processing treatment on those parameters [20,21,22,23,24,25,26] have been reported in previous studies. Even though the majority of the phenolic components are widely reported to bind to the fibre matrix of grains, little is known about the chemical composition and antioxidant activities of the phenolics in rice bran dietary fibre. The differences in the phenolic profiles of rice bran and its soluble and insoluble dietary fibres are also unclear. The bioaccessibility of phenolic compounds in foods is a crucial factor affecting their biological activity, however, the bioaccessibility of the phenolics in dietary fibre has not been reported in the literature. Even though the bioaccessibility of the phenolic compounds in brown rice has been reported [27], significant differences in the chemical composition and the digestibility of the dietary fibre of rice bran and brown rice may lead to different release rates of the phenolic compounds during digestion. Therefore, the objectives of this study are to (1) compare the chemical composition and antioxidant activity of the phenolics in rice bran and its soluble and insoluble dietary fibres and (2) compare the bioaccessibility of the phenolic compounds in rice bran and its soluble and insoluble dietary fibres.

## 2. Results and Discussion

### 2.1. Phenolic and Flavonoid Contents

The free, bound and total phenolic and flavonoid contents in DRB and its dietary fibres were presented in Table 1. The bound and total phenolic contents and free, bound and total flavonoid contents in SDFDRB were the lowest, whilst the free phenolic content in IDFDRB was the lowest of the 3 analysed samples. When compared with IDFDRB, DRB had more free and total phenolics but fewer bound phenolics. The results of the flavonoid contents of DRB and IDFDRB were similar to those of the phenolics.

As shown in Table 1, 65.8% and 85.3% of phenolics and flavonoids existed in free form in DRB, respectively. Some free phenolics would undoubtedly be released into water and even degraded during the gelatinization and sequential enzyme hydrolysis processes, which could account for the obvious reduction in the free phytochemical content in IDFDRB compared to that of DRB. The retention of a small quantity of free phenolics and flavonoids was attributed to physical blocking and noncovalent binding of the fibrous matrix. Similarly, previous studies reported that free phenolics are more susceptible to losses due to hydrothermal treatment [28,29]. In contrast, the solvent nonextractable bound phytochemicals were believed to be more stable at high temperatures since they are covalently bound to macromolecules such as fibre and protein [30,31]. Due to the removal of starch and protein from the DRB, the content of bound phenolics and flavonoids in IDFDRB was increased from 263.6 mg GAE/100 g DW and 52.8 mg CE/100 g DW to 607.1 mg GAE/100 g DW and 158.2 mg CE/100 g DW, respectively. The percentage contribution of bound phenolics and flavonoids to the total was therefore increased from 34.2% and 14.7% to 90.0% and 58.3%, respectively. The removal of starch and protein also caused the SDFDRB had a lower content of free phenolics and flavonoids, whilst the percentage contributions of bound phenolics and flavonoids to the total in SDFDRB was 6.3% and 33.8% higher than those of DRB, respectively. Based on the yields of SDF and IDF from DRB (2.5%, 28.9%, respectively), it could be deduced that the majority of the bound phenolics in DRB was conjugated to IDFDRB.

### 2.2. Phenolic Composition

Seventeen monomeric phenolic compounds were detected in DRB and its dietary fibres, namely, gallic acid, protocatechuic acid, chlorogenic acid, *p*-hydroxybenzonic acid, catechin, vanillic acid, caffeic acid, syringic acid, epicatechin, vanilline, *p*-coumaric acid, ferulic acid, sinapic acid, isoquercitrin, caffeic acid methyl, quercetin, and ferulic acid methyl. Additionally, four unidentified substances were deduced to be phenolic compounds according to their UV spectral data. Specifically, peak 3 may be a phenolic acid. The content of each of the 21 monomeric phenolics and percentage contribution of free and bound fractions to the total were presented in Table 2. (Supplementary Materials Figure S1).

The phenolic composition and contents of DRB, SDFDRB and IDFDRB were significantly different. All the 21 monomeric phenolics could be detected in DRB, although there were only traces of *p*-hydroxybenzonic acid, caffeic acid, and sinapic acid present. The findings were in agreement with previous reports [26,32]. For the dietary fibres, sinapic acid was not found in SDFDRB, and gallic acid, protocatechuic acid, chlorogenic acid, catechin, caffeic acid, epicatechin, and sinapic acid were not found in IDFDRB. Only 8 out of the 21 detected compounds, namely, vanillic acid, *p*-coumaric acid, ferulic acid, isoquercitrin and four unidentified substances, were present in DRB and both its dietary fibres.

The unidentified phenolic acid (peak 3) was the primary free monomeric phenolic compound in DRB, while vanilline was the predominant free monomeric phenolic compound in SDFDRB and IDFDRB. Similarly, vanilline was the predominant free monomeric phenolic compound in foxtail millet dietary fibre [33]. Conversely, ferulic acid and *p*-coumaric acid were the major bound monomeric phenolic compounds in DRB and IDFDRB, and they totally accounted for 72.1% and 74.1% of their total bound monomeric phenolics, respectively. Similar results were found in earlier studies on rice bran [17,18]. However, vanillic acid [34,35] and hydroxybenzonic acid [15] were reported to be the major bound monomeric phenolics together with the predominant ferulic acid in rice bran. The discrepancies among these studies might be attributed to the differences in rice varieties, cultivating environments, etc. Ferulic acid was the major bound monomeric phenolic compound in SDFDRB, accounting for 70.3% of the total content of bound monomeric phenolics. Furthermore, more than 95% of the ferulic acid present in DRB, SDFDRB and IDFDRB was in bound form. Similar case was also reported in SDF and IDF of red rice [36]. Conversely, gallic acid and hesperidin were the major bound phenolic acids in mango peel dietary fibre [37] and citrus (*Citrus junos* Sieb. ex Tanaka) insoluble dietary fibre [38], respectively. Hence, the composition of bound phenolics in dietary fibre is mainly related to the source of the fibre.

The total bound monomeric phenolics measured by HPLC method accounted for 87.8%, 82.1% and 98.6% of the total content of monomeric phenolics in DRB, SDFDRB and IDFDRB, respectively. However, based on the Folin-Ciocalteu colorimetric method, bound phenolics accounted for 34.2%, 40.5% and 90.0% of the contents of total phenolics in DRB, SDFDRB and IDFDRB, respectively. Many factors could partly explain this discrepancy among them, the complexity of the components of the bound fraction extracts and the nonspecificity of the Folin-Ciocalteu colorimetric method might be the major factors. The bound phenolic contents in DRB, SDFDRB and IDFDRB measured by the 2 methods were consistent with each other, but that was not the case for the free phenolic contents. The free phenolics in the samples were extracted with aqueous organic solvent. Inevitably, some reducing components, such as amino acids and reducing sugars, as well as ascorbic acid, could be extracted along with the free phenolics and react with Folin-Ciocalteu reagent, resulting in an over-estimation of the value [39,40]. Furthermore, owing to the dearth of essential technical data and standard substances, we failed to quantify those unidentified substances in free phenolics in DRB, SDFDRB and IDFDRB, resulting in an under-estimation of the value by the HPLC method. Additionally, due to its solubility, some SDFDRB itself may dissolve in the solvent when its free phenolics were extracted, which would lead to an over-estimation of the value by the Folin-Ciocalteu colourimetric method.

Apart from phenolic acids, 4 monomeric flavonoids, namely, catechin, epicatechin, isoquercitrin and quercetin, were also detected. There was a measurable amount of each of the 4 monomeric flavonoids in DRB and SDFDRB, but only trace or undetectable levels of these compounds in IDFDRB. Despite the possible presence of other unidentified flavonoids, phenolic acids are likely the predominant phenolic compounds bound to IDFDRB, which is in agreement with the results of other studies [11,32,36]. Some flavonoids bound to proteins or SDF in DRB may be removed during the enzymatic hydrolysis of the protein and the removal of SDF during the preparation of IDFDRB. Taken together, there were significant differences in both the phenolic composition and contents among DRB and its fibres.

### 2.3. Antioxidant Capacity by FRAP, ORAC and CAA

To better evaluate the potential antioxidant properties of DRB and its fibres, three assays, namely, FRAP (ferric reducing antioxidant power), ORAC (oxygen radical absorbance capacity) and CAA (cellular antioxidant activity) assays, were carried out in the present study. The obtained results were presented in Table 3.

As for the free fractions, DRB had the highest antioxidant capacity among the FRAP, ORAC and CAA assays followed by SDFDRB and IDFDRB. These results were in accordance with the phenolic contents among DRB and its fibres (Table 1). Furthermore, vanilline may be the major contributor to the antioxidant activity due to its high proportion in the free fractions of DRB and its fibres (Table 2). With regard to the bound fractions, IDFDRB showed the highest antioxidant activity by all 3 assays, followed by DRB and SDFDRB, which was in agreement with its higher content of bound phenolics. Presumably, *p*-coumaric acid and ferulic acid are the major contributors to the antioxidant activity due to their high proportion in the bound fraction of IDFDRB (Table 2). As with the total antioxidant activity, DRB possessed the highest FRAP and ORAC values, IDFDRB showed the highest CAA activity, and SDFDRB had the lowest values in all 3 assays.

As shown in Table 3, the FRAP value of the free fractions contributed more than 70% of the total FRAP value of DRB, but only 10% for IDFDRB. The ORAC values of the free fractions contributed more than 60% of the total ORAC value of DRB, but less than 40% and 10% to SDFDRB and IDFDRB, respectively. In contrast, the CAA values of the bound fractions contributed more than 70%, 80% and 90% of the total CAA values of DRB, SDFDRB and IDFDRB, respectively. Hence, the antioxidant activity of IDFDRB was mostly determined by its bound phenolics. Conversely, the free phenolics were the major contributors to the antioxidant activity (DPPH radical scavenging capacity and FRAP) of insoluble dietary fibre from citrus (*Citrus junos* Sieb. ex Tanaka) [38]. This discrepancy may be due to the difference in the phenolic composition and contents. The total antioxidnt activities of IDFDRB as evaluated by these three methods were 2–4 times greater than those of SDFDRB. Similarly, the IDF from red rice showed a DPPH radical scavenging activity nearly 17 times greater than that of the corresponding SDF [36]. 

### 2.4. Bioaccessibility of the Phenolic Compounds in Rice Bran and Its Dietary Fibres

Above data proved that DRB and its dietary fibres were rich in phenolic compounds. However, the consumption of foods containing abundant phenolic compounds does not usually mean that a substantial amount of active compounds will be absorbed by the human body [41,42]. The biological properties of phenolics may mostly depend on their release from the food matrix during digestion, which determines the bioaccessibility of the phenolic compounds. The bioaccessibility of a nutrient can be defined as the percentage of the nutrient released from the food matrix making it available for absorption through gastrointestinal mucosa after digestion [43]. Phenolic bioaccessibility directly influences the in vivo physiological function of these compounds. To date, there have been few studies involving the phenolic bioaccessibility of DRB and its dietary fibres. Hence, the bioaccessibility of the phenolic compounds in DRB and its dietary fibres were investigated in this study.

Considering rice is usually consumed after being cooked, DBR was cooked by steaming for 20 min prior to being subjected to in vitro digestive tests. As shown in Table 4, the release of the phenolics in DRB and IDFDRB mainly occurred in the stomach, whereas the phenolics in SDFDRB were mainly released in the small intestine. The highest content of bioaccessible phenolics (461.7 mg GAE/100 g DW) after gastrointestinal digestion was observed in DRB followed by SDFDRB (188.1 mg GAE/100 g DW). Meanwhile, the highest phenolic bioaccessibility (87.4%) was found in SDFDRB followed by DRB (60.0%). IDFDRB had the least bioaccessible phenolics (126.9 mg GAE/100 g DW) and the lowest phenolic bioaccessibility (18.8%). These results were consistent with previous studies [44,45] that found that the SDF/IDF ratio in cereal products is positively correlated to their phenolic bioaccessibility. The phenolic bioaccessibility of IDFDRB was also much lower than those of nuts (56.87%), vegetables (26.01%), legumes (25.47%) and fruits (40.77%) and seven grains (Sorghum arundinaceum, Eleusinecorocana, Sorghum bicolor (red variety), Amaranthus hybridus, Rottboelliacochinchinensis, Panicum maximum and Brachiariabrizantha, average, 28%) [46,47]. To our best knowledge, there have been no reports involving the phenolic bioaccessibility of dietary fibre from other materials.

Phenolic bioaccessibility are mostly determined by the existing form of the phenolic compounds. In fact, the higher free phenolic proportions in the above mentioned food materials accounted for their higher phenolic bioaccessibility. Free phenolics are compartmentalized primarily in vacuoles in the plant cells, whilst bound phenolics are mainly covalently bound to structural carbohydrates and proteins of the cell walls. During the digestion of food material, most of the free phenolics are released in the upper digestive tract by hydrolyzation by gut enzymes, whilst the bound phenolics are mostly released in the lower digestive tract by hydrolysation by a combination of gut enzymes and microbial enzymes [21,31]. Many researchers have reported that phenolics entering the lower digestive tract can have health benefits. A portion of the released phenolics are converted into lower molecular weight phenolics or metabolites that are subsequently absorbed into the blood through the epithelial cells of the gastrointestinal tract and then exert health benefits, however, some other phenolic compounds may contribute to maintaining a healthy, antioxidant environment by scavenging free radicals or offsetting the effects of dietary pro-oxidants together with the detained phenolics in the residue from food materials after digestion [4,37,48,49]. The low phenolic bioaccessibility of IDFDRB was mainly attributed to its higher proportion of bound phenolics. Hence, IDFDRB may exert important health benefits in the lower digestive tract.

## 3. Materials and Methods

### 3.1. Chemical and Reagents

Heat-stable α-amylase (Termamyl 120 L, 120 KNU-S/mL), protease (Alcalase 2.4 L, 4.42 AU/mL), and amyloglucosidase (AMG 300 L, 300 AGU/mL) were purchased from Novozymes (Beijing, China) biotechnology Co., Ltd. Folin—Ciocalteu reagent, 6-hydroxy-2,5,7,8-tetramethylchroman-2-carboxylic acid (Trolox), 2,4,6-tripyridyl-s-triazine (TPTZ), 3′,6′-dihydroxy-spiro[isobenzofuran-1(^3^H),9′-(^9^H)-xanthene]-3-one disodium salt (FL) and 2,2′-azobis-(2-amidinopropane) dihydrochloride (ABAP) were purchased from Sigma Chemical Co. (St. Louis, MO, USA). Gallic acid, chlorogenic acid, protocatechuic acid, *p*-hydroxybenzonic acid, vanillic acid, catechin, epicatechin, caffeic acid, sinapic acid, syringic acid, vanilline, isoquercitrin, *p*-coumaric acid, ferulic acid, quercetin, caffeic acid methyl, ferulic acid methyl were purchased from Aladdin Reagents (Shanghai, China). Fetal bovine serum (FBS), Hanks’ balanced salt solution (HBSS) and Dulbecco’s modified eagle’s medium (DMEM) were purchased from Atlanta Biologicals (Lawrenceville, GA, USA), GibcoLife Technologies (Grand Island, NY, USA) and Thermo Fisher Scientific (Waltham, MA, USA), respectively. HepG2 human liver cancer cells were purchased from the American Type Culture Collection (ATCC) (Rockville, MD, USA). All other chemicals used were of analytical grade or above.

### 3.2. Rice Grain Samples and Sample Preparation

The rice grains used in this study were obtained from the experimental farm of Guangdong Academy of Agricultural Sciences, China. The rice grains were grown in the 2015 season, harvested directly from fields in July 2016, and air-dried until their moisture content was reduced to approximately 12%. Then, they were dehusked and polished using a Satake Rice Machine (Satake Co., Hiroshima, Japan) to obtain approximately 10% (*w*/*w*) of fresh rice bran. Freshly prepared rice bran was sieved by passage through an 80-mesh sieve and stored at −20 °C until analysis.

### 3.3. Preparation of the Defatted Rice Bran and Its Dietary Fibres

Defatted rice bran (DRB) was obtained by extracting fresh rice bran three times with 10-volume hexane at ambient temperature for 24 h under constant shaking to remove lipids. Freshly prepared DRB was ground and sieved by passage through an 80-mesh sieve. The soluble dietary fibre from DRB (SDFDRB) and insoluble dietary fibre from DRB (IDFDRB) were prepared based on the following procedures. Briefly, 60 g of DRB was mixed with 600 mL of distilled water and gelatinized at 95 °C for 10 min. Gelatinized DRB was subjected to sequential enzymatic digestion with α-amylase (1.8 mL, pH 6.0, 95 °C, 20 min), alcalase protease (3.0 mL, pH 7.5, 60 °C, 60 min) and amyloglucosidase (1.2 mL, pH 4.5, 60 °C, 30 min) in a water bath to remove the starch and protein. After centrifugation (6800× *g*, 10 min), the residue was washed twice with distilled water at 60 °C, and then dried at 40 °C for 24 h to yield IDFDRB. The supernatant was combined with the washings of the residue, and the mixture was gently neutralized by 10% (*m*/*v*) Ca(OH)_2_ to precipitate phytic acid. The mixture was then dialyzed (10 kDa molecular weight cut-off (MWCO) membrane) at 25 °C for 24 h to desalt. The fraction failed to dialysis was concentrated to 1/4 of its original volume at 45 °C and precipitated by adding 4-volume ethanol overnight. It was then freeze-dried in a FDU-2110 freeze dryer (Tokyo physical and Chemical Corporation) for 72 h to yield SDFDRB. The dried materials were ground into a fine powder to pass an 80-mesh sieve. All samples were stored at −20 °C until analysis.

### 3.4. Chemical Composition

Samples were analysed in triplicate for their moisture, starch, fat, crude protein (N × 5.95) and ash contents according to AOAC (2005) methods 934.01 (gravimetric method), 978.10 (enzyme hydrolysis method), 948.22 (using aSoxhlet apparatus and petroleum ether), 960.52 (Kjeldahl method) and 942.05 (incineration at 525 °C), respectively. Chemical composition of DRB and its dietary fibres can be seen in Table 5.

### 3.5. Extraction of Free Phenolics

The free phenolic compounds were extracted by the method reported previously [50] with some modifications. Briefly, 1.0 g of DRB or its dietary fibre was blended with 50 mL of chilled 80% acetone at 10,000 rpm for 5 min in an ice bath. Then, the supernatant was separated by centrifugation at 8000× *g* for 10 min, and the extraction procedure was repeated once. The two supernatants were combined and evaporated at 45 °C under vacuum and reconstituted to a final volume of 10 mL with chilled MeOH. The solution was then stored at −20 °C until further analysis.

### 3.6. Extraction of Bound Phenolics

Bound phenolics were extracted by the method reported in previous studies [51]. Briefly, the residue from the extraction of the free phenolics was digested with 40 mL of 2 M NaOH at room temperature. The mixture was continuously shaken for 1 h under a nitrogen atmosphere. Then, the mixture was neutralized with concentrated hydrochloric acid and extracted 5 times with ethyl acetate. The pooled ethyl acetate supernatants were evaporated and reconstituted with chilled MeOH to a final volume of 10 mL, and the solution was then stored at −20 °C until analysis.

### 3.7. Determination of the Phenolic Content

The phenolic content was determined using the method reported previously [52]. Briefly, 0.125 mL of the extracts was sequentially mixed with 0.5 mL of distilled water and 0.125 mL of Folin-Ciocalteu reagent. After letting the mixture react for 6 min, 1.25 mL of 7% aqueous Na_2_CO_3_ and 1.0 mL of water were sequentially added to the mixture. The final mixture was kept in the dark at room temperature for 90 min, and then its absorbance was measured at 760 nm. A standard curve was constructed by using gallic acid as the standard. The final results were expressed as mg of gallic acid equivalents (GAE) per 100 g dry weight (DW) of sample.

### 3.8. Determination of the Flavonoid Content

The flavonoid content was determined by the method reported previously [52]. Briefly, a 0.3 mL of the above extracts was sequentially mixed with 1.5 mL of distilled water and 0.09 mL of 5% NaNO_2_ solution. Six min later, 0.18 mL of 10% AlCl_3_·6H_2_O solution was added to the mixture. Five min later, 0.6 mL of 1 M NaOH solution and 0.33 mL of distilled water were sequentially added. The absorbance of the final mixture was immediately determined at 510 nm. A standard curve was constructed by using catechin as the standard. The final results were expressed as mg of catechin equivalents (CE) per 100 g dry weight (DW) of sample.

### 3.9. Determination of the Phenolic Compositions

Prior to HPLC analysis, all samples were passed through a 0.25 μm membrane filter (Millipore, Billerica, MA, USA). Phenolic compositions were measured according to the procedure reported previously [53] with some modifications. Briefly, a 20 μL aliquot was analysed using an Agilent 1260 HPLC system (Waldbronn, Germany) equipped with a DAD detector and a Zorbax SB-C18 analytical column (250 × 4.6 mm, i.d., 5 μm; Palo Alto, CA, USA). The mobile phase consisted of 0.4% aqueous acetic acid (solvent A) and acetonitrile (solvent B) at a flow rate of 1.0 mL/min. The column temperature was 25 °C. Gradient elution was performed as follows: from 0 to 40 min, linear gradient from 5% to 25% solution B; from 40 to 45 min, linear gradient from 25% to 35% solution B; from 45 to 50 min, linear gradient from 35% to 50% solution B; and from 50 to 55 min, linear gradient from 50% to 5% solution B. Simultaneous monitoring was set at 280 nm for the quantification of monomeric phenolics. Based on the previous reports [17,18,19,26,32,34,35,36,53], the phenolic compounds in the samples were identified by comparing their experimental retention times and UV spectral data with those of known authentic standards.

### 3.10. Antioxidant Activity Determined by FRAP Assay

The FRAP assay was carried out according to the method reported previously [54]. The working solution was freshly prepared by mixing 25 mL of 300 mM acetate buffer (3.1 g of CH_3_COONa·3H_2_O and 16 mL of CH_3_COOH per litre of buffer solution, pH 3.6), 2.5 mL of 10 mM TPTZ solution (3.12 g TPTZ per litre of 40 mM HCl), and 2.5 mL of 20 mM FeCl_3_·6H_2_O solution and incubating the solution at 37 °C for 10 min prior to use. A 0.09 mL of the diluted extract was vigorously mixed with 2.0 mL of the above working solution for 1 min and then placed in the dark for 60 min. The absorbance was then measured at 593 nm. Trolox was used to prepare a standard curve, and the FRAP antioxidant activity was expressed as μmol of Trolox equivalents per g DW of sample.

### 3.11. Antioxidant Activity Determined by ORAC Assay

The ORAC assay was carried out by the method reported previously [55]. The determination was performed using black-walled 96-well plates. Each well was sequentially charged with 20 μL of diluted extracts or Trolox standard at concentrations ranging from 6.25 to 50 μM and 200 μL of fluorescein (final concentration 0.96 μM). The mixtures were incubated at 37 °C for 20 min, and then 20 μL of 119 mM ABAP was added to each well. Finally, the fluorescence intensity was measured using a Fluoroskan Ascent FL plate-reader (Tecan Infinite 200, Männedorf, Austria) at an excitation wavelength of 485 nm and an emission wavelength of 538 nm every 4.5 min for a total of 35 measurements. The results were expressed as μmol Trolox equivalents per g DW of sample.

### 3.12. Antioxidant Activity Determined by CAA Assay

Prior to the CAA determination, the phenolic extracts were evaporated and reconstituted with isopyknic distilled water. The CAA assay was performed according to the method previously reported [56]. Briefly, HepG2 cells were inoculated at a density of 6 × 10^4^ per well in a 96-well black-walled microplate in 100 μL of growth medium (DMEM containing 10% FBS), and cultivated in a CO_2_ incubator at 37 °C for 24 h. After removing the medium and rinsing with PBS, the seeded wells were treated for 1 h with DMEM containing 25 μM DCFH-DA with or without phenolic extracts. Subsequently, 600 μM ABAP in 100 μL of HBSS was added to each well except for the blank wells. The fluorescence of each well (excitation at 485 nm, emission at 538 nm) was measured by a Fluoroskan Ascent FL plate reader (Tecan Infinite 200, Männedorf, Austria) every 5 min for a total of 12 measurements. The results were reported as μmol quercetin equivalents (QE) per 100 g DW of sample.

### 3.13. Bioaccessibility of the Phenolic Compounds in Different Samples

The bioaccessible phenolics were determined by an in vitro gastrointestinal digestion method following a previously reported procedure [57] with some modifications. Briefly, 1.5 g of DRB or its dietary fibre was placed in a 100 mL vessel, mixed with 30 mL of distilled water and heated in a water bath at 37 °C. A 1.5 mL aliquot of pepsin (20 g/L in 0.1 M HCl) was then added into the vessel, and the pH was adjusted to 2.0 with 6 M HCl. The gastric digestion was carried out for 1 h. The pH of the mixture was then adjusted to 7.2 with 1 M NaHCO_3_ to stop gastric digestion. Intestinal digestion was initiated by sequentially adding 7.5 mL of pancreatin/bile solution (2 g/L of pancreatin and 12 g/L of bile salt in 0.1 M NaHCO_3_) and 7.5 mL of KCl/NaCl (120 mM NaCl and 5 mM KCl), and digestion was continued for 2.5 h. The supernatant was obtained by centrifugation at 6000× *g* for 10 min at 4 °C and then purified by precipitation by adding 4-volume ethanol to remove SDF. The phenolic bioaccessibility was calculated as the percentage of bioaccessible phenolics present in the purified supernatant relative to the total phenolic content in DRB or its dietary fibre. A blank (without added DRB or its dietary fibre) was incubated under the same conditions, and its phenolic content was determined to correct for interferences from the digestion processes. The vessel was magnetically stirred during all the digestion processes.

### 3.14. Statistical Analyses

All treatments were conducted in triplicate, and the results were reported as the mean ± SD. Statistical analysis was carried out using the IBM SPSS Statistic Version 20.0 software. Data were analysed by ANOVA, and the differences between means were assessed by Dunnett’s tests or *t* tests. A value of *p*< 0.05 was considered to be significantly different. During the analysis of phenolic composition, when a compound could be detected only in 2 of the 3 samples, the *t*-test was used to evaluate the statistical difference between the contents.

## 4. Conclusions

This study revealed that IDFDRB had a higher proportion of bound phenolics and higher CAA activity than DRB and SDFDRB. However, IDFDRB showed lower phenolic bioaccessibility than DRB and SDFDRB. Bound phenolics are likely released from food matrix in the colon by the action of gut microbiota and may exert significant beneficial health effect in vivo. The release of the phenolics from IDFDRB in vivo and the related health effects require further investigation.

## Figures and Tables

**Table 1:** Phenolic and flavonoid contents of defatted rice bran and its dietary fibres.

	DRB	SDFDRB	IDFDRB
*Phenolics* ^A^			
Free	506.1 ± 7.5a * (65.8) **	128.0 ± 5.5b (59.5)	67.4 ± 0.4c (10.0)
Bound	263.6 ± 9.8b (34.2)	87.3 ± 0.6c (40.5)	607.1 ± 0.3a (90.0)
Total	769.7 ± 17.2a	215.3 ± 5.1c	674.6 ± 0.5b
*Flavonoids* ^B^			
Free	305.3 ± 11.7a (85.3)	10.2 ± 0.2c (51.5)	113.1 ± 2.8b (41.7)
Bound	52.8 ± 5.6b (14.7)	9.6 ± 0.2c (48.5)	158.2 ± 2.4a (58.3)
Total	358.1 ± 7.2a	19.8 ± 0.4c	271.3 ± 0.5b

DRB, defatted rice bran. SDFDRB, soluble dietary fibre from DRB. IDFDRB, insoluble dietary fibre from DRB. * Values in the same line with no letters in common are significantly different among different samples (*p* < 0.05). ** Values in parentheses indicate percentage contribution to the total content. ^A^ mg GAE/100 g DW. ^B^ mg CE/100 g DW.

**Table 2:** Phenolic profiles of defatted rice bran and its dietary fibres (μg/g DW).

Phenolics	Varieties	Free Form	Bound Form	Total
Gallic acid	DRB	5.1 ± 0.5 * (100)	nd	5.1 ± 0.5 *
	SDFDRB	2.4 ± 0.0 (100)	nd	2.4 ± 0.0
	IDFDRB	nd	nd	nd
Protocatechuic acid	DRB	11.1 ± 0.4 (100)	nd	11.1 ± 0.4
	SDFDRB	nd	0.9 ± 0.0 (100)	0.9 ± 0.0
	IDFDRB	nd	nd	nd
Unknown (Peak 3)	DRB	138.0 ± 4.9a (100)	nd	138.0 ± 4.9a
	SDFDRB	7.6 ± 0.3b (100)	nd	7.6 ± 0.3b
	IDFDRB	5.5 ± 0.2c (100)	nd	5.5 ± 0.2c
Chlorogenic acid	DRB	10.4 ± 1.4 * (100)	nd	10.4 ± 1.4 *
	SDFDRB	6.1 ± 0.0 (100)	nd	6.1 ± 0.0
	IDFDRB	nd	nd	nd
*p*-Hydroxybenzonic acid	DRB	Tr	Tr	Tr
	SDFDRB	nd	0.2 ± 0.0 (100)	0.2 ± 0.0
	IDFDRB	nd	2.9 ± 0.1 * (100)	2.9 ± 0.1 *
Catechin	DRB	26.0 ± 1.5 * (65.4)	12.9 ± 1.7 (34.6)	39.8 ± 2.6 *
	SDFDRB	3.4 ± 0.1 (100)	nd	3.4 ± 0.1
	IDFDRB	nd	nd	nd
Vanillic acid	DRB	15.8 ± 2.1a (62.2)	9.3 ± 0.6b (37.8)	25.5 ± 2.1a
	SDFDRB	1.5 ± 0.0b (33.9)	2.9 ± 0.1c (66.1)	4.4 ± 0.1c
	IDFDRB	1.3 ± 0.0c (9.9)	11.3 ± 0.3a (90.1)	12.6 ± 0.3b
Caffeic acid	DRB	Tr	nd	Tr
	SDFDRB	0.3 ± 0.0 (15.8)	1.6 ± 0.0 (84.2)	1.8 ± 0.0
	IDFDRB	nd	nd	nd
Syringic acid	DRB	nd	3.7 ± 0.4b (100)	3.7 ± 0.4b
	SDFDRB	0.4 ± 0.0 (23.5)	1.2 ± 0.0c (76.5)	1.5 ± 0.0c
	IDFDRB	nd	4.8 ± 0.0a (100)	4.8 ± 0.0a
Epicatechin	DRB	79.7 ± 7.4 * (60.9)	50.8 ± 1.0 * (39.1)	130.8 ± 8.5 *
	SDFDRB	11.0 ± 0.3 (28.2)	28.1 ± 0.3 (71.8)	39.1 ± 0.6
	IDFDRB	nd	nd	nd
Vanilline	DRB	88.1 ± 4.9b (26.7)	242.0 ± 12.6 * (73.3)	330.0 ± 12.9a
	SDFDRB	60.2 ± 3.1c (84.1)	11.4 ± 0.2 (15.9)	71.5 ± 2.9c
	IDFDRB	103.0 ± 2.4a (100)	nd	103.0 ± 2.4b
*p*-Coumaric acid	DRB	13.5 ± 0.9a (3.3)	402.4 ± 53.5b (96.8)	416.0 ± 54.0b
	SDFDRB	4.8 ± 0.0b (22.9)	16.0 ± 0.5c (77.1)	20.8 ± 0.5c
	IDFDRB	2.90 ± 0.1c (0.3)	1098.6 ± 5.2a (99.7)	1101.5 ± 5.2a
Ferulic acid	DRB	26.1 ± 2.8a (1.4)	1784.9 ± 131.8b (98.6)	1810.9 ± 129.0b
	SDFDRB	12.3 ± 0.0b (3.2)	373.6 ± 5.0c (96.8)	385.9 ± 5.0c
	IDFDRB	2.4 ± 0.2c (0.1)	5124.3 ± 39.4a (99.9)	5126.6 ± 39.2a
Sinapic acid	DRB	Tr	nd	Tr
	SDFDRB	nd	nd	nd
	IDFDRB	nd	nd	nd
Isoquercitrin	DRB	Tr	20.9 ± 2.5 * (100)	20.9 ± 2.5 *
	SDFDRB	Tr	0.11 ± 0.0 (100)	0.11 ± 0.0
	IDFDRB	Tr	Tr	Tr
Caffeic acid methylester	DRB	2.1 ± 0.2 * (20.6)	8.4 ± 0.7a (79.4)	10.3 ± 0.6a
	SDFDRB	2.0 ± 0.0 (82.4)	0.4 ± 0.0c (17.6)	2.4 ± 0.0c
	IDFDRB	nd	5.2 ± 0.1b (100)	5.2 ± 0.1b
Unknown (Peak 17)	DRB	nd	42.4 ± 1.1b (100)	42.4 ± 1.1b
	SDFDRB	nd	9.2 ± 0.4c (100)	9.2 ± 0.4c
	IDFDRB	nd	362.6 ± 10.8a (100)	362.6 ± 10.8a
Unknown (Peak 18)	DRB	nd	472.9 ± 11.4b (100)	472.9 ± 11.4b
	SDFDRB	nd	77.9 ± 2.3c (100)	77.9 ± 2.3c
	IDFDRB	nd	1553.8 ± 40.7a (100)	1553.8 ± 40.7a
Quercetin	DRB	Tr	0.7 ± 0.1 * (100)	0.7 ± 0.1 *
	SDFDRB	nd	0.3 ± 0.0 (100)	0.3 ± 0.0
	IDFDRB	Tr	Tr	Tr
Unknown (Peak 20)	DRB	5.5 ± 0.2a (8.9)	56.6 ± 2.6b (91.1)	62.1 ± 2.3b
	SDFDRB	3.8 ± 0.1c (35.5)	6.9 ± 0.3c (64.5)	10.7 ± 0.2c
	IDFDRB	4.5 ± 0.2b (1.9)	236.3 ± 7.2a (88.1)	240.8 ± 6.5a
Ferulic acid methylester	DRB	0.3 ± 0.0(27.5)	0.7 ± 0.2b(72.5)	0.9 ± 0.2b
	SDFDRB	Tr	0.6 ± 0.0b (100)	0.6 ± 0.0c
	IDFDRB	nd	2.00 ± 0.1a (100)	2.0 ± 0.1a
Total monomeric phenolics	DRB	422.3 ± 20.8a (12.2)	3032.0 ± 100.9b (87.8)	3454.4 ± 98.7b
	SDFDRB	115.6 ± 19.2b (17.9)	531.2 ± 5.5c (82.1)	646.8 ± 18.6c
	IDFDRB	119.6 ± 2.2b (1.4)	8401.7 ± 128.3a (98.6)	8521.3 ± 127.4a

DRB, defatted rice bran. SDFDRB, soluble dietary fibre from DRB. IDFDRB, insoluble dietary fibre from DRB. The unidentified phenolic compounds (peak 3, 17, 18, 20) were caculated as ferulic acid equivalent. Tr, Trace; nd, not detectable. Values with different letters or * in each column are significantly different among different samples (*p* < 0.05). Values in parentheses indicate percentage contribution to the total content.

**Table 3:** Antioxidant activities of defatted rice bran and its dietary fibres.

Samples	Free	Bound	Total
*FRAP* ^A^			
DRB	34.7 ± 0.1a * (72.0) **	13.8 ± 0.3b (28.0)	48.5 ± 0.3a
SDFDRB	4.1 ± 0.1b (47.0)	4.6 ± 0.1c (53.0)	8.7 ± 0.2c
IDFDRB	3.7 ± 0.0c (12.0)	27.9 ± 0.1a (88.0)	31.6 ± 0.2b
*ORAC* ^B^			
DRB	228.5 ± 15.7a (67.4)	110.7 ± 2.4b (32.6)	339.1 ± 6.7a
SDFDRB	23.0 ± 0.3b (32.6)	47.5 ± 0.5c (67.4)	70.6 ± 0.8c
IDFDRB	17.2 ± 1.1b (8.9)	178.9 ± 8.7a (91.1)	196.4 ± 10.0b
*CAA* ^C^			
DRB	31.7 ± 2.2a (22.9)	106.7 ± 14.2b (77.1)	138.5 ± 12.2b
SDFDRB	12.3 ± 0.2b (15.7)	66.0 ± 0.4c (84.3)	78.3 ± 0.3c
IDFDRB	3.0 ± 0.1c (1.2)	248.5 ± 0.6a (98.8)	251.3 ± 0.3a

DRB, defatted rice bran. SDFDRB, soluble dietary fibre from DRB. IDFDRB, insoluble dietary fibre from DRB. * Values in the same column with no letters in common are significantly different among different samples (*p* < 0.05). ** Values in parentheses indicate percentage contribution to the total. ^A^ μmol TE/g DW. ^B^ μmol TE/g DW. ^C^ μmol QE/100 g DW.

**Table 4:** Phenolic bioaccessibility of defatted rice bran and its dietary fibres.

Samples	Total Phenlic Content (mg GAE/100 g DW)	Bioaccessible Phenolics after GD (mg GAE/100 g DW)	Bioaccessible Phenolics after GID (mg GAE/100 g DW)
DRB	769.7 ± 17.2a *	443.2 ± 22.5a (57.6) **	461.7 ± 24.9a (60.0)
SDFDRB	215.3 ± 5.1c	67.0. ± 0.7c (31.1)	188.1 ± 0.9b (87.4)
IDFDRB	674.6 ± 0.5b	92.2 ± 0.2b (13.7)	126.9 ± 0.5c (18.8)

DRB, defatted rice bran. SDFDRB, soluble dietary fibre from DRB. IDFDRB, insoluble dietary fibre from DRB. GD, gastric digestion. GID, gastrointestinal digestion. Total phenol content was calculated as the sum of free and bound phenolics. * Values in the same column with no letters in common are significantly different among different samples (*p* < 0.05). ** Values in parentheses indicate the phenolic bioaccessibility, which was calculated as the percentage of bioaccessible phenolics after GD or GID to the total phenolics in samples.

**Table 5:** Chemical composition of defatted rice bran and its dietary fibres.

	DRB	SDFDRB	IDFDRB
Water (%)	13.0 ± 0.2	nd	11.0 ± 0.1
Starch (%)	30.5 ± 1.2	2.8 ± 0.0	4.9 ± 0.0
Fat (%)	Tr	nd	nd
Protein (%)	13.8 ± 0.4	12.2 ± 0.2	9.8 ± 0.2
Ash (%)	10.1 ± 0.3	8.6 ± 0.1	4.5 ± 0.1

DRB, defatted rice bran. SDFDRB, soluble dietary fibre from DRB. IDFDRB, insoluble dietary fibre from DRB. nd, not detectable. Tr, Trace.

## Supplementary Materials

Supplementary materials are available online.
