# Supplementary material for: A Comparison of the Chemical Composition, In Vitro Bioaccessibility and Antioxidant Activity of Phenolic Compounds from Rice Bran and Its Dietary Fibres
Source: Molecules. 2018 Jan 18;23(1):202. doi: 10.3390/molecules23010202 (PMC6017774; doi:10.3390/molecules23010202)
Supplement: Supplementary file 1 [file molecules-23-00202-s001.pdf]

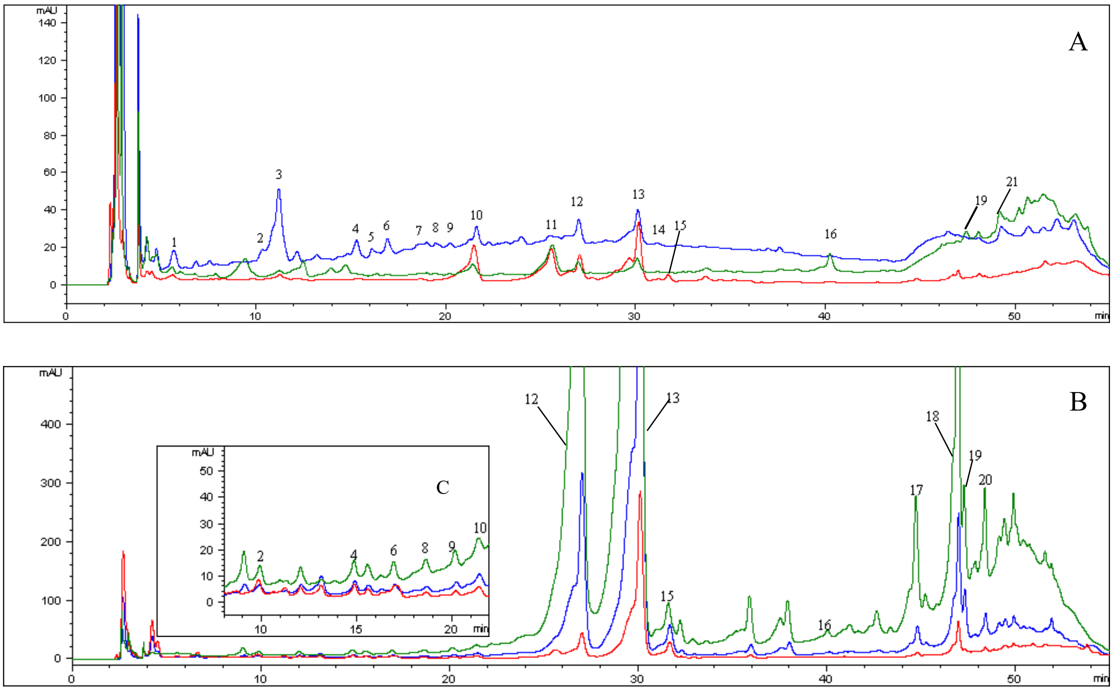

Figure S1. HPLC chromatogram of free phenolic extract (A) and bound phenolic extract (B, C) of DRB (blue), SDFDRB (red) and IDFDRB (green) (280 nm): (1), gallic acid, (2), protocatechuic acid, (3), unidentified phenolic acid, (4), chlorogenic acid, (5), *p*-hydroxybenzoic acid, (6), catechin , (7), vanillic acid, (8), caffeic acid, (9), syringic acid, (10), epicatechin, (11), vanillin, (12), *p*-coumaric acid, (13), ferulic acid, (14), sinapic acid, (15), isoquercitrin, (16), caffeic acid methyl ester, (17), unidentified phenolic compound, (18), unidentified phenolic compound, (19), quercetin, (20), unidentified phenolic compound, (21), ferulic acid methyl ester.
